# A Multi-Laboratory Comparison of Methods for Detection and Quantification of African Swine Fever Virus

**DOI:** 10.3390/pathogens11030325

**Published:** 2022-03-07

**Authors:** Ann Sofie Olesen, Thomas Bruun Rasmussen, Søren Saxmose Nielsen, Graham J. Belsham, Anette Boklund, Tosca Ploegaert, Bernie Moonen-Leusen, Sandra Blome, Anette Bøtner

**Affiliations:** 1 DTU National Veterinary Institute, Technical University of Denmark, Lindholm, DK-4771 Kalvehave, Denmark; asjo@ssi.dk (A.S.O.); tbru@ssi.dk (T.B.R.); grbe@sund.ku.dk (G.J.B.); 2 Section of Veterinary Clinical Microbiology, Department of Veterinary and Animal Sciences, University of Copenhagen, DK-1870 Frederiksberg C, Denmark; 3 Department of Virus & Microbiological Special Diagnostics, Statens Serum Institut, DK-2300 Copenhagen S, Denmark; 4 Section of Animal Welfare and Disease Control, Department of Veterinary and Animal Sciences, University of Copenhagen, DK-1870 Frederiksberg C, Denmark; saxmose@sund.ku.dk (S.S.N.); anebo@sund.ku.dk (A.B.); 5 Department of Virology, Wageningen Bioveterinary Research, Wageningen University & Research, 8221 RA Lelystad, The Netherlands; tosca.ploegaert@wur.nl (T.P.); bernie.moonen@wur.nl (B.M.-L.); 6 Institute of Diagnostic Virology, Friedrich-Loeffler-Institut, Insel Riems, 17493 Greifswald, Germany; sandra.blome@fli.de

**Keywords:** African swine fever, qPCR, quantification, ring trial, virus infectivity, virus titration

## Abstract

African swine fever is a viral disease of the family *Suidae*. Methods to detect and quantify African swine fever virus (ASFV) include qPCR and virus infectivity assays. Individual laboratories often use in-house procedures for these assays, which can hamper the comparison of results. The objective of this study was to estimate the probability of ASFV detection using these assays, and to determine the inter-test correlations between results. This was achieved by testing a panel of 80 samples at three reference laboratories. Samples were analysed using nucleic acid extraction and qPCR, as well as virus infectivity assays. For qPCR, a very high probability (ranging from 0.96 to 1.0) of detecting ASFV DNA was observed for all tested systems. For virus infectivity assays in cells, the probability of detecting infectious ASFV varied from 0.68 to 0.90 and was highest using pulmonary alveolar macrophages, followed by MARC145 cells, peripheral blood monocytes, and finally wild boar lung cells. Intraclass correlation coefficient estimates of 0.97 (0.96–0.98) between qPCR methods, 0.80 (0.74–0.85) to 0.94 (0.92–0.96) between virus infectivity assays, and 0.77 (0.68–0.83) to 0.95 (0.93–0.96) between qPCR methods and virus infectivity assays were obtained. These findings show that qPCR gives the highest probability for the detection of ASFV.

## 1. Introduction

African swine fever (ASF) is an infectious haemorrhagic disease of the family *Suidae*, including domestic pigs and wild boar. It is caused by African swine fever virus (ASFV), a large double-stranded DNA virus, the only member of the *Asfivirus* genus within the *Asfarviridae* family [1]. ASF has largely been restricted to Africa for most of its history, but spread to countries outside of Africa (e.g., in Europe, South America, and Cuba) has occurred [2]. The latest epidemic outside of the African continent started in Georgia in 2007, and ASFV has since spread to neighbouring countries, e.g., Russia, and also further to the West (within Europe and recently to the Americas) and East (into Asia and the Pacific) [3]. Given the serious socioeconomic threat that the disease poses to major pig-producing countries, ASF is considered a highly important (re)emerging disease of global concern. ASFV is difficult to grow in established cell cultures without prior adaptation. Thus, wild type viruses (e.g., samples from animal studies using ASFV, and field samples of ASFVs from natural outbreaks) have frequently been grown in certain primary porcine cells (i.e., peripheral blood monocytes or alveolar macrophages). Primary cells are, however, difficult to obtain in large quantities and often exhibit batch-to-batch variation, hampering the reproducibility of the results [4,5]. Certain ASFVs have been shown to grow well in an African green monkey kidney-derived established cell line, i.e., VERO cells, following virus adaptation [4,5,6]. More recently, some studies have also described the use of other monkey-derived cell lines to grow ASFVs from natural outbreaks or from experiments, namely COS-1 cells [7], MARC145 cells [8], and MA-104 cells [9]. In other studies, porcine-derived cell lines, e.g., WSL cells (derived from wild boar lungs, [10,11]), the immortalized porcine alveolar monocyte/macrophage (IPAM) cell line [12], Zuckerman macrophage-4 (ZMAC-4) cells (derived from foetal pig lung, [13]), and the immortalized porcine kidney macrophages (IPKMs) [14] have been shown to support the growth of wild type ASFVs (i.e., not just cell-culture-adapted strains). In order to assess virus infection of cells, the haemadsorption assay [15] and various immuno-detection systems [16,17] have been applied. The results of these tests are commonly presented as either the 50% haemadsorption dose (HAD_50_/_mL_) or the 50% tissue culture infectious dose (TCID_50_/_mL_).

In recent years, quantitative real-time polymerase chain reaction (qPCR) assays have been widely used to detect and quantify virus genomic DNA as a marker for the virus [18,19,20]. Results obtained from qPCR assays can be reported semi-quantitatively using Cq-values or TCID_50_-equivalents. The TCID_50_-equivalent values do not represent the amount of infectious virus but are a measure of the relative levels of viral DNA in a sample [8]. The use of a calibration curve with known concentrations of the target DNA (e.g., within a plasmid) allows for an absolute quantification of the DNA fragment targeted by the primers and the probe used in the qPCR [21].

While individual laboratories may have carried out comparisons of different methodologies to a certain extent, the published levels of ASFV DNA or infectious ASFV are often difficult to compare between laboratories and even between individual experiments. This may hamper the proper interpretation and comparison of results from both experimental and field studies.

The primary objective of this study was to estimate the probability of ASFV detection and the inter-test correlation between qPCR and virus infectivity assays in cells within different laboratories using their own in-house systems. The participating laboratories were national reference laboratories for ASFV and involved in ASFV diagnostics, surveillance, and research.

## 2. Results

### 2.1. Detection and Quantification of Viral DNA by qPCR

**Figure 1:**
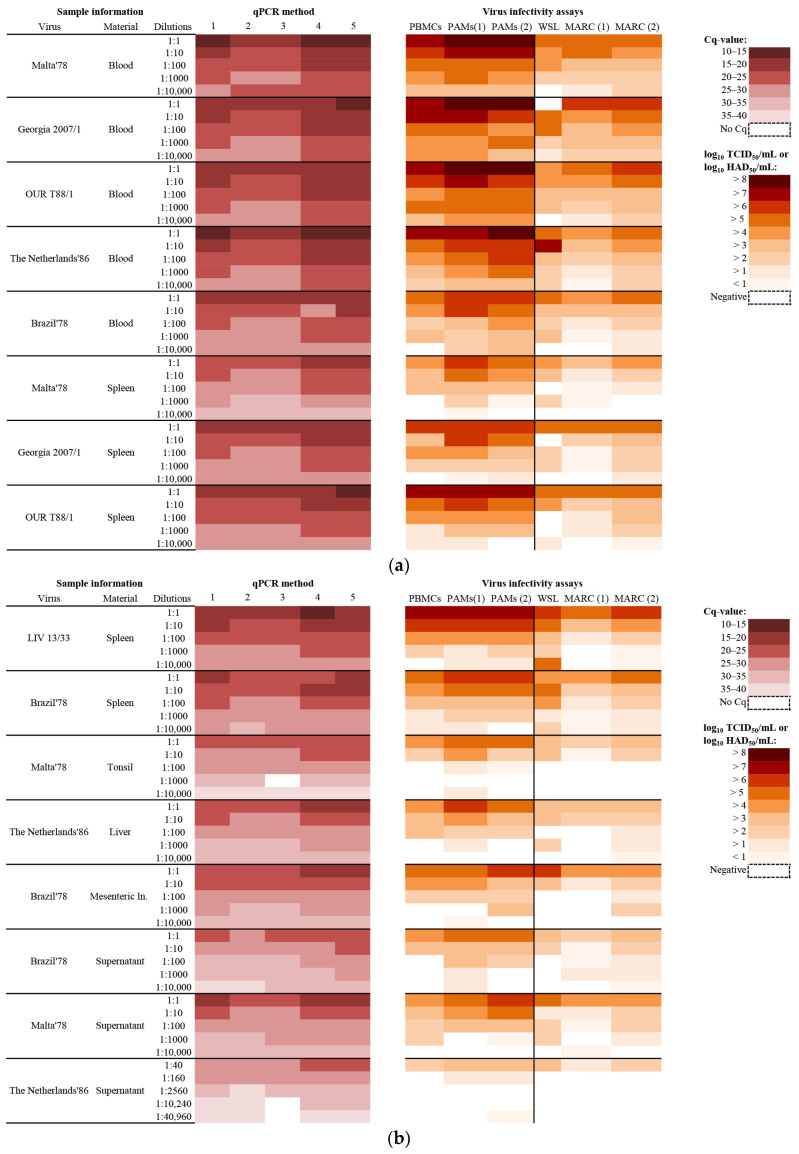
(**a**). Heat map based on Cq-values and virus titres for samples 1–40. (**b**) Heat map based on Cq-values and virus titres for samples 41–80.

The Cq-values obtained using five different methods for the extraction and detection of viral DNA (from now on referred to as the five qPCR methods) in the three laboratories are illustrated as heat maps in Figure 1a,b. The actual Cq-values are provided in Table S1. The log_10_TCID_50_-equivalents (from Methods 4 and 5) and the standard curves used for calculation of these equivalents are shown in Table S2 and Figure S1. The absolute genome copy numbers (from Method 1) and the standard curve used to determine them are provided in Table S3 and Figure S2.

The probabilities of detecting ASFV DNA using each qPCR method (including the individual calculations for replicates from Methods 1, 4, and 5) are shown in Table 1. In general, the detection of ASFV DNA by the different methods was very efficient. Detection probabilities just below 1.0 (0.96, 0.98, and 0.99) were obtained for three methods (Methods 3, 4, and 5, see Table 1). However, for Methods 4 and 5, the probability of detecting ASFV DNA was still assessed to be 1.0 when analysing technical replicates (Table 1). False negative results were obtained for five samples in total (samples 30, 54, 78, 79, and 80). The samples were all highly diluted (see Figure 1a,b). Cq-values from 29.9 to 38.5 were detected in these samples (Table S1).

ln = lymph node, PBMCs = peripheral blood mononuclear cells, PAMs = pulmonary alveolar macrophages, WSL = wild boar lung cells, MARC = MARC145 cells, (1) virus infectivity assay performed at WBVR, (2) virus infectivity assay performed at DTU, St. = standard curve samples. Virus titres are reported as log_10_ HAD_50_/mL for the PBMC assay and the PAM assay at WBVR. Titres for the remaining four assays are reported as log_10_ TCID_50_/mL. qPCR results from Methods 1, 4, and 5 and the virus titration results from MARC145 (DTU) are shown as the mean of technical replicates. Mean values were calculated after the conversion of Cq-values or virus titres (on a log_2_-scale and log_10_-scale) to the direct scale through exponentiation. Cq-values and titres (including values for each of the technical replicates) are shown in Tables S1 and S4.

The intraclass correlation coefficient (ICC) for the Cq-values obtained by the five qPCR methods was calculated as 0.97 (0.96–0.98), indicating a high level of agreement between the obtained Cq-values and an excellent reliability (as defined by [22]). The Cq-values within each method were found to follow a Normal distribution.

### 2.2. Detection and Quantification of Virus Infectivity in Cells

The virus infectivity titres obtained from titrations in primary cells or using cell lines at the three laboratories are illustrated as heat maps in Figure 1a,b. The actual titres are provided in Table S4.

The highest virus titres were obtained using the primary cell assays, while the established cell lines displayed lower sensitivities (Figure 1a,b, Table S4). Amongst the three different primary cell assays, the highest virus titres were determined, for the majority of the samples, using PAM assays. For the cell line assays, the highest titres were obtained for approximately half of the samples using the MARC145 cell assay, and for the other samples using the WSL cell assay (Figure 1a,b, Table S4).The estimated probabilities of detecting ASFV in each virus infectivity assay (including individual calculations for the replicates in MARC145 cells at DTU) are shown in Table 1. The probability of detecting infectious ASFV was highest when using PAMs (84% to 90%), then progressively lower with MARC145 cells (74% to 85%), PBMCs (75%), and finally WSL cells (0.68) (Table 1). For five samples, no infectious virus was detected using any of the three primary cell assays. Low levels of infectious virus were detected in two of these five samples (samples 60 and 75) when they were inoculated onto MARC145 cells (Figure 1a,b, Table S4). Samples 60 and 75 were highly diluted liver and cell supernatant samples (from PAMs), respectively (Figure 1b).

In nine samples, no infectious virus was detected using any of these three cell line assays. However, infectious virus was detected in six of these nine samples (samples 30, 53, 55, 65, 77, and 80), when they were inoculated onto primary cells (Figure 1a,b, Table S4). Samples 30, 53, 55, and 65 were medium to highly diluted organ samples (spleen, tonsil, or lymph nodes), while samples 77 and 80 were medium to highly diluted cell supernatant samples (from PAMs) (Figure 1a,b).

The ICC estimate for the match between the titres obtained using the six virus infectivity assays was 0.88 (95% confidence interval: 0.84–0.91), which corresponds to a good level of reliability [22]. For the virus titres obtained using only the three primary cell assays, an ICC of 0.94 (0.92–0.96) was estimated (corresponding to excellent reliability), while for the three cell line assays and the two MARC145 cell assays, ICCs of 0.80 (0.74–0.85) and 0.93 (0.89–0.95) were estimated, respectively. These indicate a higher level of agreement when the same type of cells (porcine primary cells of the monocyte-macrophage lineage or MARC145 cells, respectively) were used for the assays. Titres within each assay were found to follow a Normal distribution. 

### 2.3. Comparison between qPCR and Virus Infectivity Assays

ICC estimates generated for the relationship between the viral genome quantities (genome copies or TCID_50_ equivalents) and the virus titres are shown in Table 2. ICC estimates ranged from 0.77 (0.68–0.83) to 0.95 (0.93–0.96) (Table 2). Viral genome quantities and titres within each method and assay were deemed to follow a Normal distribution.

**Figure 2:**
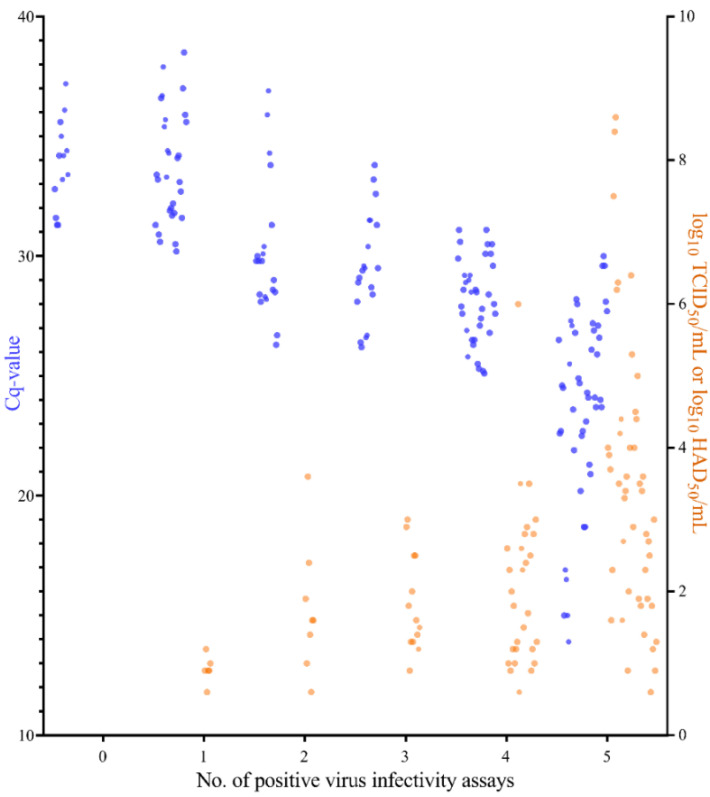
Sample titres (right *y*-axis) and Cq-values (left *y*-axis) for samples in which infectious virus was detected by only some or none of the virus infectivity assays. Blue dots = Cq-values, orange dots = titres (log_10_ HAD_50_/mL, or log_10_ TCID_50_/mL).

The Cq-values and infectivity titres obtained for the 33 samples, in which no infectious virus was detected by some or all of the six virus infectivity assays, are depicted as a function of the number of “positive” virus infectivity assays in Figure 2. The 33 samples included blood, organ (liver, spleen, lymph node, tonsil) homogenate, and cell culture supernatant samples. As expected, the number of “positive” virus infectivity assays increased when the sample titres were higher and the observed sample Cq-values decreased. Samples in which no infectious virus was detected in either assay, or in only one assay, had Cq-values varying from 29.9 to 37.2 (no “positive” virus infectivity assays), or 29.6 to 38.5 (one “positive” virus infectivity assay) (Figure 2). Using the plasmid standard curve used for absolute quantification at DTU, these Cq-values corresponded to approximately 2–4.5 log_10_ genome copies/mL. This means that the virus infectivity assays can be expected to fail (or be unreliable) if the Cq-value is >30, or less than about 1000 virus genomes/mL are present in the sample (see also Section 3).

## 3. Discussion

In this study, in-house methods for nucleic acid extraction followed by qPCR, and in-house virus infectivity assay procedures for the detection and quantification of ASFV were compared in three different national reference laboratories. Using qPCR, viral DNA was detected in all 80 samples that were known to contain virus. The probability of detecting ASFV DNA was high for all the applied qPCR methods, with most of the methods obtaining an ASFV detection probability of 100% (Table 1). The false negative results reported for five samples by one or more methods occurred with highly diluted samples (1:1000 or more), e.g., samples 79 and 80 (Figure 1b, Table S1), i.e., the amount of viral DNA was close to the detection limit of the applied qPCR method. Detection limits for the five qPCR methods have been reported to be 10–100 molecules/3 µL ([18]—Method 3) or 5.7–57 copies/5 µL ([20]—Methods 1 and 2). By absolute quantification based on the pVP72 standard curve at DTU (Method 1), a Cq-value of 40 has been calculated to correspond to the detection of approximately 1 (0.5) viral genome copy per qPCR input amount (5 µL) [23]; this means that a Cq-value of 30 would correspond to the presence of approximately 1000 genome copies/5 µL (50,000 genome copies/mL blood). Using the same approach for the highly diluted samples 79 and 80, the samples demonstrated viral genome copy numbers of about 126–501 genome copies/mL (Table S3), corresponding to about 2.5–10 genome copies/5 µL. This is within the range of the reported detection threshold for Method 1 (see above).

Besides the detection limits of the applied methods, instrument or operator errors could also cause false negative results.

For comparison of the five qPCR methods, the ICC estimate and its associated 95% confidence intervals indicated an excellent correlation between the obtained Cq-values [22]. This suggests that, even though different procedures were used for nucleic acid extraction and qPCR (e.g., different instruments, reporter dyes, etc.) at the three reference laboratories, the results obtained for the samples were very consistent for the five different laboratory methods.

For the virus infectivity assays, the highest ASFV titres were obtained using primary cell assays and only lower titres were found using the established cell line assays. These findings indicate that primary cells are the most suitable for the detection of the non-cell culture adapted (wild type) ASFVs used in this study (as described previously, [4,5]). For more efficient replication in cell lines, adaptation to a particular cell line is usually needed; for example, at DTU, it has previously been found that VERO-adapted Ba71V cells can grow to high titres in another monkey-derived cell line, MARC145 [24]. The Ba71V strain was obtained by adaptation of the highly virulent Ba71 strain, isolated from spleen material from pigs in Badajoz in 1971, to grow in VERO cells. It has been reported that this cell-culture adapted strain is non-pathogenic for pigs [25]. In the present study, it was observed that ASFV titres determined using the PAM assay were generally higher than those obtained in the PBMC assay (Figure 1a,b, Table S4). This might be due to differences in the maturation stage of these primary cells in the respective assays. Previously, the susceptibility of cells of the porcine monocyte-macrophage lineage to ASFV infection has been investigated [26]. These authors tested cells representing three maturation stages, namely bone marrow precursor cells, blood monocytes, and alveolar macrophages. For both virulent and attenuated ASFVs (wild type ASFV E75 or ASFV E75 attenuated by passage in cell culture), the susceptibility to infection increased as the cells matured, i.e., alveolar macrophages were more susceptible to infection than blood monocytes. Even when monocytes were matured in vitro to monocyte-derived macrophages, infection levels were lower when compared to alveolar macrophages [26]. Similar results have been obtained by others, who observed that monocyte differentiation into macrophages increased cell susceptibility to ASFV infection [27].

In the PAM assays, as used at DTU and WBVR, virus detection was based on either haemadsorption or immunostaining assays. Similar titres were reported for these two detection methods (Figure 1a,b, Table S4), indicating that, when measuring ASFV infectivity, one HAD_50_ corresponds to approximately one TCID_50_. Haemadsorption assays have the advantage that the plates can be read repeatedly on a daily basis, while immunostaining only allows the plates to be read at a single time point. However, non-haemadsorbing ASFVs, which have been reported from Europe [28], would be missed when applying an assay relying on virus detection by haemadsorption.

In this study, the probability of detecting infectious ASFV was the highest using PAM assays, followed by assays with MARC145 cells, PBMCs, and then WSL cells. The probabilities for detecting infectious ASFV using the MARC145 cell assays (74% to 85%) were comparable to those for PAMs (84% to 90%) and PBMCs (75%), indicating that the MARC145 cells are suitable for virus detection. Even though primary cells seem more appropriate for virus amplification and most likely virus detection in samples with a low initial amount of infectious virus (as higher titres were obtained using primary cells), the issues of reproducibility and supply [4,5], together with animal welfare concerns, make cell lines, such as the MARC145, a valid alternative for large scale experiments such as the titrations of samples from animal experiments. For comparison of the virus infectivity assays, the ICC estimates and their associated 95% confidence intervals indicated good to excellent (all six assays), excellent (primary cell assays), moderate to good (all cell line assays), and good to excellent (MARC145 cell assays) reliabilities between the obtained titres [22]. Titres obtained using the same type of cells (e.g., primary cells of the monocyte/macrophage lineage, or the same type of cell line) yielded ICC estimates that were comparable to those obtained when comparing the five qPCR methods.

However, a lower correlation between titres when compared to Cq-values would be expected due to several factors, including differences in the sensitivity of the cells, cell densities, amount of inoculum, incubation conditions, and staining procedures (Table 5). In addition, variation contributed by the operator is likely to be of higher importance for virus infectivity assays compared to qPCR, as the plates are read manually using a microscope. This implies that the experience of the operator is even more crucial when compared to analysis by qPCR. For the comparison of quantification by qPCR and virus infectivity assays, the ICC estimates and their associated 95% confidence intervals indicated moderate to good, up to excellent reliabilities (see also Table 2). These findings suggest that the reported levels of ASFV DNA can, to some extent, be associated with the reported levels of infectious ASFV—depending on the assays and methods used. The relationship between the level of viral DNA and the infectious virus titre also depends on how the material has been stored before testing, since storage, e.g., at high temperature or under dry conditions, may lead to inactivation of the virus which is expected to influence the result of virus isolation. It will, however, not necessarily influence the qPCR assay, which also detects inactivated virus.

For samples in which no infectious virus was detectable by all or in only one of the virus infectivity assays, the Cq-values corresponded to approximately log_10_ 2–4.5 genome copies/mL. This suggests that around 100–30,000 genome copies need to be present in the sample volume tested to detect virus infectivity and gives an indication of the possible range of a particle to TCID_50_/HAD_50_ ratio. The suggested number of genome copies needed to detect ASFV infectivity in vitro falls in between the range of viral particles required to detect infectivity in a plaque assay (to form one plaque forming unit, PFU) for two alphaherpesviruses, namely Herpes simplex virus 1 (HSV-1) and Varicella-zoster virus (VZV) (these are also large DNA viruses but from a different virus family). For HSV-1, ratios of 14 complete particles/PFU [29] and 9–132 genomes/PFU (depending on treatment and virus strain) [30] have been reported. In contrast, for VZV, a ratio of 40,000 particles/PFU has been reported [31]. Note that, for ASFV, the minimum infectious dose in vivo may not reflect the minimum infectious dose in cells. For example, earlier studies have shown residual ASFV infectivity for pigs, in heat-treated virus stocks [32] or meat products [33] in which no infectious virus had previously been reported by virus isolation in cells. In addition, in some cases the material containing the virus could be in a form (e.g., highly contaminated) in which virus infectivity assays are not possible/reliable, even though the material maintains infectiousness in pigs [34,35].

Our findings show that qPCR gives the highest probability for detection of the virus (viral DNA) and is a very reliable method to correlate the levels of ASFV detected in different laboratories. For the virus infectivity assays, the probability of detecting ASFV was lower when compared to that of the qPCR assays. However, it should be noted that only the virus infectivity assays were able to show whether the genomes detected by the qPCR were present within infectious or non-infectious forms of the virus. For the comparison of virus infectivity, titre levels detected using assays with the same cell types at different laboratories were comparable to the same degree as reported for the interlaboratory comparison of ASFV DNA levels using qPCR assays.

## 4. Materials and Methods

### 4.1. Viruses

The ASFV test panel was assembled and distributed by the WBVR (Lelystad, the Netherlands) to two additional laboratories. Thus, three separate laboratories participated in the study. The panel contained six different strains (five within genotype I and one within genotype II) of ASFVs isolated from different geographical locations (Table 3). It consisted of dilution series of defibrinated blood samples, organ homogenates (10% *w*/*v*), and supernatants from ASFV-infected cells, resulting in 80 samples in total (Figure 1a,b). The supernatants from ASFV-infected cells were derived from virus propagated on porcine pulmonary alveolar macrophages (PAMs) and dilutions were made with RPMI 1640 (Gibco, ThermoFisher Scientific, Waltham, MA, USA) supplemented with 10% foetal calf serum (Sigma Aldrich, St. Louis, MO, USA) and 1% penicillin/streptomycin (Gibco).

### 4.2. Quantitative Real-Time Polymerase Chain Reaction (qPCR)

The three laboratories (DTU, FLI, and WBVR, i.e., the Danish, German, and Dutch National ASFV Reference Laboratories, respectively) performed DNA extraction and qPCR using in-house procedures and equipment (Table 4). The qPCR results are presented as the threshold cycle (Cq) values of either one or two technical replicates. If two replicates were produced, the mean Cq-value was used for statistical analysis of the inter-test correlation. Mean values were calculated after the conversion of Cq-values (which are essentially on a log_2_-scale) to the direct scale through exponentiation.

#### 4.2.1. DTU

Nucleic acids were extracted from the samples using a MagNA Pure 96 system (Roche) and tested for the presence of ASFV DNA by qPCR using the Mx3005P qPCR system (Agilent Technologies), essentially as described by [20] (see Table 4). P72 target amplification was identified from 6-carboxyfluorescein (FAM) dye emission. A positive result in the qPCR was determined by identification of the Cq-value at which FAM dye emission appeared above background within 40 cycles. Absolute quantification was used to determine the number of genome copies by reference to a standard curve based on a 10-fold dilution series of the plasmid, pVP72, as described by [18]. Genome copy numbers are presented as log_10_ genome copies/mL. Mean genome copy numbers of technical replicates were used for statistical analysis of the inter-test correlation. Mean values were calculated after the conversion of genome copy values (on a log10-scale) to the direct scale through exponentiation.

#### 4.2.2. FLI

Nucleic acids were extracted using the QIAamp Viral RNA Mini Kit (Qiagen) according to the manufacturer’s instructions. The kit is designed for the extraction of viral RNA. However, it was validated at the German NRL for ASFV genome detection and is routinely used to extract ASFV samples from both experimental trials and diagnostic investigations. Subsequently, qPCR was performed according to the protocols published by [18,20] with slight modifications on the C1000^TM^ thermal cycler with the CFX96TM Real-Time System (Bio-Rad). P72 target amplification was identified from 6-carboxyfluorescein (FAM) dye emission. A positive result in the qPCR was determined by identification of the Cq-value at which FAM dye emission appeared above background within 45 cycles.

#### 4.2.3. WBVR

Nucleic acids were extracted from the samples using a MagNA Pure 96 system (Roche) and tested for the presence of ASFV DNA by qPCR using the LightCycler480 qPCR system (Roche) essentially as described in [8]. P72 target amplification was identified from 6-carboxyfluorescein (FAM) dye emission. A positive result in the qPCR was determined by identification of the Cq-value at which FAM dye emission appeared above background within 40 cycles. TCID_50_-equivalents were calculated using a standard curve consisting of five dilutions (1:40, 1:160, 1:2560, 1:10,240, and 1:40,960) of The Netherlands’86 ASFV reference strain (Figure 1). TCID_50_-equivalents are presented as log_10_ TCID_50_-equivalents/mL. Mean TCID_50_-equivalents of technical replicates were used for statistical analysis of the inter-test correlation. Mean values were calculated after the conversion of TCID_50_-equivalents/mL (on a log10-scale) to the direct scale through exponentiation.

### 4.3. Virus Infectivity Assays in Cells

Virus titres of the samples were determined by end-point titration in both primary cells and cell lines at all three laboratories. Primary cells were either PAMs or peripheral blood mononuclear cells (PBMCs), and the established cell lines were wild boar lung (WSL) or MARC145 cells (Table 5). The virus infectivity results are presented as titres (log_10_ TCID_50_/mL or log_10_ HAD_50_/mL) of either one or two technical replicates. If two replicates were produced, the mean titre was used for statistical analysis of the inter-test correlation. Mean values were calculated after the conversion of virus titres (on a log10-scale) to the direct scale through exponentiation.

#### 4.3.1. DTU

PAMs were prepared as described previously [43] and suspended (at a final concentration of 2 × 10^6^ cells/mL) in Eagle’s Minimum Essential Medium (EMEM) supplemented with antibiotics (streptomycin and neomycin, Sigma-Aldrich) and 5% foetal calf serum. MARC145 cells were thawed and suspended (at a final concentration of 4 × 10^5^ cells/mL) in EMEM with 10% foetal calf serum. For end-point titration, the cells were seeded into Nunc^TM^ 96-well (U Bottom) plates (Thermo Fisher Scientific) in aliquots of 100 μL. Cells were inoculated (in triplicate) with five-fold dilutions of the samples immediately following seeding (MARC145), or one hour after seeding (PAMs). The plates were incubated for two (MARC145 cells) or three (PAMs) days at 37 °C in an atmosphere with 5% CO_2_. Following incubation, the infected cells were detected after fixation and staining of the cells using an immunoperoxidase monolayer assay (IPMA), essentially as described by [43]. Briefly, the cells were stained using an anti-ASFV antibody-positive swine serum, protein A-conjugated horseradish peroxidase (Sigma-Aldrich), and hydrogen peroxide. Stained cells were counted manually using a light microscope and ASFV titres were calculated as log_10_ TCID_50_/mL using the Reed and Muench method [44].

#### 4.3.2. FLI

Blood for the preparation of PBMC-derived macrophages was collected from domestic donor pigs as previously described [45]. To detect ASFV in the respective samples, a standard haemadsorption assay (HAD) was carried out. In brief, 100 µL of a PBMC preparation (5 × 10^6^ cells/mL) was seeded into 96-well microplates. After 16–24 h, non-adherent cells were removed and the cell culture medium containing GM-CSF was replenished (100 µL). The cells were then incubated for 24 to 48 h to allow the initial maturation of the macrophages. Subsequently, ten-fold dilution series of all samples were added, in quadruplicate, using 100 µL per well. After another 24 h, 20 µL of a 1% erythrocyte suspension originating from the same donor pig was added. For the following three days, all cultures were examined for haemadsorption. Titres were calculated using the Reed and Muench method [44].

#### 4.3.3. WBVR

PAMs from pigs of different ages were prepared as described previously [46], with minor modifications. Cells were suspended at a final concentration of 10^6^ cells/mL in RPMI1640 medium supplemented with antibiotics (1% penicillin and streptomycin, Gibco) and 10% fetal calf serum. MARC145 cells were thawed and suspended (at a final concentration of 2.5 × 10^5^ cells/mL) in DMEM^glutamax^ with antibiotics (1% penicillin and streptomycin, Gibco) and 5% fetal calf serum. For end-point titration, PAMs were seeded into Costar^TM^ 24-well plates (Corning, NY, USA) in aliquots of 1 mL. MARC145 cells were seeded into Costar^TM^ 96-well plates (Corning) in aliquots of 100 μL. Cells were inoculated with ten-fold dilutions of the samples immediately following seeding. Then, an aliquot (80 µL) of a 1% pig erythrocyte suspension was added to each well of the PAM cultures. The plates were incubated for two (MARC145 cells) or seven (PAMs) days at 37 °C in an atmosphere with 5% CO_2_. Following incubation, infected MARC145 cells were detected following fixation and staining of the cells using an immunoperoxidase monolayer assay (IPMA), slightly adapted from [46]. Briefly, the cells were stained using an anti-ASFV antibody positive swine serum, monoclonal anti-swine IgG-conjugated horseradish peroxidase (WBVR), and 3-amino-9-ethylcarbazole (AEC) with hydrogen peroxide. Stained cells were counted manually using a light microscope. Infected PAMs were visualized by rosette formation with the erythrocytes. Titres of ASFV were calculated as log_10_ TCID_50_/mL using the method described by [44].

### 4.4. Statistical Analysis

The obtained results were described using descriptive statistics with histograms for visual assessment of distributional patterns and heat maps for illustration of Cq-values and virus titres. The probabilities of detecting ASFV, using the five different combinations of nucleic acid extraction and qPCR methods and the six different virus infectivity assays, were defined as the proportions of samples containing ASFV that tested positive for viral DNA or infectious virus. The 80 samples were all derived from material known to contain the virus (viral DNA and infectious virus, see Materials and Methods). Intraclass correlation coefficients (ICC) estimates and associated 95% confidence intervals were calculated for the various methods and assays using the ICC()-function in the psych package [47] in R [48]—based on a single-rater measurement, consistency, two-way mixed-effects model (ICC 3,1). ICC values below 0.5 are defined as being indicative of poor reliability, ICC values between 0.5 and 0.75 as indicative of moderate reliability, ICC values between 0.75 and 0.9 as indicative of good reliability, and ICC values above 0.90 as indicative of excellent reliability [22].

## Figures and Tables

**Table 1:** Probability of detection ASFV by the different qPCR methods and virus infectivity assays (including technical replicates).

Test	Method/Assay	Laboratory	TP	FN	Probability of Detecting ASFV %
qPCR	1	DTU	80	0	100
	1	DTU	80	0	100
	2	FLI	80	0	100
	3	FLI	77	3	96
	4	WBVR	79	1	99
	4	WBVR	80	0	100
	5	WBVR	78	2	98
	5	WBVR	80	0	100
VI	PBMCs	FLI	60	20	75
	PAMs	WBVR	72	8	90
	PAMs	DTU	67	13	84
	WSL	FLI	54	26	68
	MARC145	WBVR	59	21	74
	MARC145	DTU	63	17	79
	MARC145	DTU	68	12	85

TP = true positive, FN = false negative, VI = virus infectivity, PBMCs = peripheral blood mononuclear cells, PAMs = pulmonary alveolar macrophages, WSL = wild boar lung cells.

**Table 2:** ICC estimates and their confidence intervals for comparison of virus detection using qPCR (genome copies for Method 1 and TCID_50_ equivalents for Methods 4 and 5) and virus infectivity assays.

VI Assay Cell Type	qPCR Quantification Method		
	Method 1 (DTU)	Method 4 (WBVR)	Method 5 (WBVR)
PBMCs (FLI)	0.93 (0.90–0.95)	0.82 (0.75–0.87)	0.86 (0.81–0.90)
PAMs (WBVR)	0.91 (0.87–0.93)	0.79 (0.71–0.85)	0.81 (0.74–0.87)
PAMs (DTU)	0.91 (0.87–0.93)	0.79 (0.71–0.85)	0.85 (0.79–0.90)
WSL (FLI)	0.82 (0.75–0.87)	0.77 (0.68–0.83)	0.80 (0.73–0.86)
MARC (WBVR)	0.91 (0.87–0.94)	0.80 (0.72–0.86)	0.86 (0.80–0.90)
MARC (DTU)	0.95 (0.93–0.96)	0.89 (0.84–0.92)	0.89 (0.84–0.92)
**Legend**			
excellent (ICC > 0.90)			
good to excellent (ICC 0.75–0.90)			
good (ICC 0.75–0.90)			
moderate to good (ICC < 0.75–0.90)			

VI = virus infectivity, PBMCs = peripheral blood mononuclear cells, PAMs = pulmonary alveolar macrophages, WSL = wild boar lung cells, MARC = MARC145 cells.

**Table 3:** African swine fever viruses in the test panel.

Virus	Country of Origin	Species of Origin	P72 Genotype	Reference
Brazil’78	Brazil	*Suidae*	I	[36]
Georgia 2007/1	Georgia 2007/1	*Suidae*	II	[37]
LIV 13/33	Zambia	*Ornithodorus*	I	[38,39]
Malta’78	Malta	*Suidae*	I	[40]
Netherlands’86	The Netherlands	*Suidae*	I	[41]
OUR T88/1	Portugal	*Ornithodorus*	I	[42]

**Table 4:** Applied in-house procedures and equipment for DNA extraction and qPCR analysis.

	DTU Method 1	FLI Method 2	FLI Method 3	WBVR Method 4	WBVR Method 5
Extraction					
Input	200 µL	140 µL	140 µL	200 µL	200 µL
Extraction kit	MagNA Pure 96 DNA/Viral NA S.V. 2.0 Kit (Roche, Basel, Switzerland)	QIAamp Viral RNA Mini Kit (Qiagen, Hilden, Germany)	QIAamp Viral RNA Mini Kit (Qiagen)	MagNA Pure LC Total Nucleic Acid Isolation Kit (Roche)	MagNA Pure LC Total Nucleic Acid Isolation Kit (Roche)
Platform	MagNA Pure 96 instrument (Roche)	na	na	MagNA Pure LC instrument (Roche)	MagNA Pure LC instrument (Roche)
Protocol	Viral NA Plasma extern lysis S.V. 3.1.	na	na	na	na
Elution volume	50 µL	50 µL	50 µL	100 µL	100 µL
**qPCR**					
Input	5 µL	5 µL	5 µL	10 µL	10 µL
Primers	ASF-P72 (1)	*ASF-P72* (1)	*ASF-P72* (2)	ASFV-p72p3 and ASFV-p72p4 (3)	ASFV-p72p3 and ASFV-p72p4 (3)
Probes	ASF-P72-FAM (1)	*ASF-P72-FAM* (1)	*ASF-P72-FAM* (2)	ASFV-p72 LC-FL and ASFV-p72 LC-Red640 (3)	ASFV-p72 LC-FL and ASFV-p72 LC-Red640 (3)
PCR kit	QuantiTect^TM^ Multiplex PCR kit (Qiagen) (1)	*QuantiTect^TM^ Multiplex PCR kit (Qiagen)* (1)	*QuantiTect^TM^ Multiplex PCR kit (Qiagen)* (2)	*DNA kit (Roche)*	*Quantifast Probe RT-PCR kit (Qiagen)*
Platform	Mx3005P qPCR system (Agilent Technologies, Santa Clara, CA, USA)	CFX 96 Real-Time System (Bio-Rad, Hercules, CA, USA)	CFX 96 Real-Time System (Bio-Rad)	LightCycler^®^ 480 (Roche)	LightCycler^®^ 480 (Roche)
Cycle conditions	2 min–15 min–1 min–1 min (last two steps 45 cycles)	15 min–1 min–1 min (last two steps 45 cycles)	15 min–1 min–1 min (last two steps 45 cycles)	10 min–1 sec–10 sec–10 sec (last three steps 45 cycles)	10 min–1 sec–10 sec–10 sec (last three steps 45 cycles)
Cycle temperature	50–95–94–60 °C	95–95–60 °C	95–95–60 °C	95–95–59–72 °C	95–95–59–72 °C

In italics = different primer, probes or PCR kits used in the same laboratory. na = not applicable, (1) as described by [20], (2) as described by [18], (3) as described by [8]. Note, the Danish National Reference Laboratory for ASFV has now been transferred from DTU to the Statens Serum Institut, Copenhagen.

**Table 5:** Applied in-house procedures for measuring virus infectivity.

	DTU	DTU	FLI	FLI	WBVR	WBVR
Cells	PAMs	MARC145	PBMCs	WSL	PAMs	MARC145
Plates	96 well	96 well	96 well	96 well	24 well	96 well
Cells/well	2 × 10^5^	4 × 10^4^	5 × 10^4^	1.5 × 10^5^	1 × 10^6^	2.1 × 10^5^
Detection method	IPMA	IPMA	HAT	IFA	HAT	IPMA
Sample amount/well	50 µL	50 µL	100 µL	100 µL	125 µL	100 µL
Incubation	2 days	3 days	3 days	3 days	3–7 days	3 days

PAMs = pulmonary alveolar macrophages, PBMCs = peripheral blood mononuclear cells, WSL = wild boar lung cells, IPMA = immunoperoxidase monolayer assay, HAT = haemadsorption test, IFA = immunofluorescence assay.

## Data Availability

The data presented in this study are available on request from the corresponding author. The raw data is available in the Supplementary Materials of this manuscript.

## Supplementary Materials

The following supporting information can be downloaded at: https://www.mdpi.com/article/10.3390/pathogens11030325/s1, Table S1: qPCR results as Cq-values; Table S2: qPCR results as log_10_ TCID_50_-equivalents; Table S3: qPCR results expressed as log_10_ genome copy numbers; Table S4: Virus infectivity assay results in different cell types; Figure S1: Standard curves applied to determine TCID_50_ equivalents (in Excel); Figure S2: Standard curve applied to determine absolute copy numbers (in MxPro).
