# Supplementary material for: A Multi-Laboratory Comparison of Methods for Detection and Quantification of African Swine Fever Virus
Source: Pathogens. 2022 Mar 7;11(3):325. doi: 10.3390/pathogens11030325 (PMC8949307; doi:10.3390/pathogens11030325)

Table S1: qPCR results as Cq-values

	Method 1 (DTU)		Method 2 (FLI)	Method 3 (FLI)	Method 4 (WBVR)		Method 5 (WBVR)	
	Cq 1	Cq 2	Cq 1	Cq 1	Cq 1	Cq 2	Cq 1	Cq 2
Sample no.								
1	14.6	14.5	18.0	17.6	11.0	13.0	12.9	11.0
2	16.5	16.5	20.7	20.4	15.7	17.0	15.8	16.8
3	21.0	21.6	23.9	23.5	17.9	19.4	18.2	19.1
4	23.9	24.2	26.6	25.9	21.2	22.7	21.3	22.4
5	26.6	26.3	22.6	22.7	23.7	25.2	23.9	24.9
6	15.1	14.9	16.9	16.5	15.0	15.0	13.0	14.4
7	18.5	18.6	21.2	21.4	15.0	17.0	16.1	17.2
8	21.2	21.6	22.3	22.4	18.0	19.9	18.4	19.6
9	23.7	23.8	25.7	25.6	21.7	22.9	21.7	22.8
10	26.2	26.2	28.2	28.3	23.2	24.6	23.2	24.6
11	16.5	17.0	17.6	17.7	18.1	16.3	17.5	16.0
12	17.2	17.2	20.0	20.0	15.2	16.7	15.5	16.5
13	20.5	20.3	23.0	23.1	17.2	18.4	17.5	18.0
14	22.8	23.0	25.2	25.4	20.0	22.0	20.7	21.7
15	25.3	25.6	27.3	27.1	22.7	24.2	22.9	17.2
16	13.7	13.6	17.8	17.3	11.0	12.0	11.5	11.0
17	17.3	17.4	21.0	20.7	15.3	16.6	15.4	16.3
18	21.1	21.8	23.4	23.0	18.3	18.6	18.3	18.6
19	23.5	24.2	25.8	25.4	20.7	22.2	20.9	22.0
20	27.4	26.6	28.6	28.1	23.9	24.9	23.7	24.9
21	18.1	18.3	19.6	19.7	15.4	16.2	16.4	16.2
22	20.8	20.4	21.5	21.7	28.1	19.1	21.8	18.7
23	24.4	24.4	25.3	25.2	21.7	22.7	21.7	22.5
24	26.6	26.8	27.7	27.7	23.8	25.6	23.9	25.2
25	27.8	28.3	28.9	29.1	25.5	26.9	25.5	26.7
26	20.4	20.6	21.6	21.0	17.2	18.7	17.1	18.4
27	23.2	23.6	25.2	25.1	20.6	22.5	20.7	22.1
28	26.5	27.0	28.2	28.0	24.0	25.5	24.0	25.2
29	29.7	30.1	31.1	30.6	26.8	28.5	26.8	28.1
30	31.4	31.2	33.4	33.2	No Cq	30.9	No Cq	30.6
31	17.1	17.5	18.3	18.3	15.0	15.8	14.0	15.8
32	20.2	20.2	22.5	22.7	17.5	19.4	18.0	19.2
33	23.9	24.1	25.6	25.3	21.4	22.8	21.3	22.6
34	26.8	26.5	27.6	27.6	24.1	25.5	24.1	25.3
35	29.5	29.3	29.6	29.5	26.0	27.0	26.2	27.0
36	16.2	16.4	18.8	18.9	20.0	15.0	15.0	14.0
37	20.5	20.3	22.4	22.5	19.9	17.8	16.9	17.7
38	22.8	23.4	24.3	24.1	20.1	21.9	20.3	21.3
39	26.0	26.1	27.2	26.9	22.9	24.8	22.0	24.5
40	28.5	28.7	29.2	28.9	25.6	27.6	25.7	25.9

41	15.8	15.3	18.8	18.7	14.0	14.0	12.0	16.3
42	18.8	19.9	21.9	21.7	16.5	17.7	16.6	18.6
43	22.3	22.6	24.2	24.1	19.9	20.8	19.9	22.1
44	25.4	26.2	27.1	26.6	23.1	24.5	23.2	24.1
45	29.0	28.9	29.2	28.5	25.2	27.2	25.4	26.9
46	18.9	19.9	20.2	20.9	22.3	17.5	17.4	17.6
47	22.1	21.6	23.2	24.1	19.0	19.7	19.2	19.5
48	24.9	25.0	26.2	26.3	21.9	23.9	22.0	23.6
49	27.7	28.5	28.1	27.9	25.0	26.3	24.9	26.1
50	29.4	29.7	30.0	29.6	26.9	28.7	26.9	28.2
51	22.6	23.3	23.4	23.7	20.6	21.8	22.3	21.6
52	26.3	26.7	27.2	27.7	24.0	25.1	24.0	24.9
53	29.9	29.7	30.0	29.8	27.6	28.9	27.4	28.6
54	32.6	33.0	31.6	No Cq	29.9	32.0	30.3	31.9
55	35.9	37.1	36.7	37.9	34.2	36.1	33.9	36.5
56	20.3	20.7	21.3	21.4	17.3	18.9	18.7	18.9
57	23.6	23.6	25.2	25.1	21.2	22.3	21.2	22.1
58	26.5	26.4	28.6	28.5	24.7	26.0	24.7	25.7
59	30.6	30.2	31.5	31.5	27.9	29.2	27.8	28.8
60	33.4	33.2	34.4	34.3	30.8	32.5	30.8	32.6
61	20.7	20.6	20.3	20.6	19.2	18.5	18.9	18.2
62	23.5	23.5	24.0	24.2	20.5	22.3	21.3	22.1
63	27.1	27.1	27.4	27.8	24.5	25.7	24.5	25.5
64	30.1	29.4	30.1	30.4	27.1	29.0	27.1	28.8
65	31.6	31.7	32.2	31.8	29.6	31.0	29.7	30.5
66	23.6	23.8	25.1	24.9	20.1	21.5	20.2	21.2
67	26.7	27.0	28.2	28.0	24.0	25.7	24.1	25.5
68	30.1	30.1	31.1	30.5	27.6	28.9	27.7	24.2
69	33.6	32.5	33.8	32.6	30.4	31.9	30.4	26.9
70	35.8	35.9	36.9	34.3	32.5	34.5	32.3	17.1
71	19.4	20.3	22.5	22.1	15.6	17.4	15.6	17.2
72	23.4	24.3	26.8	27.4	20.8	22.2	20.8	22.1
73	27.1	27.1	28.2	27.8	24.3	26.0	24.5	25.7
74	30.1	30.1	30.5	29.6	27.1	28.6	27.1	28.0
75	33.6	34.4	34.2	33.1	30.4	33.5	30.3	32.3
76 (St.)	27.1	27.0	27.0	27.0	24.0	25.2	24.0	25.0
77 (St.)	28.5	28.6	29.0	28.5	26.1	26.5	26.0	27.1
78 (St.)	33.5	34.7	35.6	35.0	31.5	33.9	No Cq	34.2
79 (St.)	36.0	36.1	37.2	No Cq	33.6	34.9	33.1	33.6
80 (St.)	35.9	37.6	38.5	No Cq	35.4	36.3	35.0	36.0

Cq = Cq-value, 1 = 1st run by the same method, 2 = 2nd run by the same method, St. = standard curve samples.

Table S2: qPCR results as log_{10} $TCID_{50}$-equivalents

	Method 4 (WBVR)		Method 5 (WBVR)	
	log_{10} $TCID_{50}$ equivalents/mL 1	log_{10} $TCID_{50}$ equivalents/mL 2	log_{10} $TCID_{50}$ equivalents/mL 1	log_{10} $TCID_{50}$ equivalents/mL 2
Sample no.				
1	7.0	6.2	6.6	7.5
2	5.8	5.3	5.9	5.9
3	5.3	4.8	5.3	5.3
4	4.4	4.1	4.4	4.4
5	3.8	3.6	3.7	3.7
6	6.0	5.7	6.6	6.5
7	6.0	5.3	5.8	5.8
8	5.2	4.7	5.2	5.2
9	4.3	4.1	4.3	4.3
10	3.9	3.7	3.9	3.8
11	5.2	5.5	5.4	6.1
12	5.9	5.4	5.9	6.0
13	5.4	5.0	5.4	5.6
14	4.7	4.3	4.6	4.6
15	4.1	3.8	4.0	5.8
16	7.0	6.4	7.0	7.5
17	5.9	5.4	6.0	6.0
18	5.2	5.0	5.2	5.4
19	4.6	4.2	4.5	4.5
20	3.8	3.7	3.8	3.8
21	5.9	5.5	5.7	6.1
22	2.7	4.9	4.3	5.4
23	4.3	4.1	4.3	4.4
24	3.8	3.5	3.8	3.7
25	3.4	3.3	3.3	3.3
26	5.4	5.0	5.5	5.5
27	4.6	4.2	4.6	4.5
28	3.7	3.5	3.7	3.7
29	3.0	2.9	3.0	2.9
30	No Cq	2.4	No Cq	2.2
31	6.0	5.6	6.3	6.2
32	5.4	4.8	5.3	5.3
33	4.4	4.1	4.4	4.4
34	3.7	3.6	3.7	3.6
35	3.2	3.2	3.1	3.2
36	4.7	5.7	6.1	6.7
37	4.8	5.2	5.6	5.7
38	4.7	4.3	4.7	4.7

39	4.0	3.7	4.2	3.9
40	3.3	3.1	3.3	3.5
41	6.2	6.0	6.9	6.0
42	5.6	5.2	5.7	5.4
43	4.8	4.5	4.8	4.5
44	4.0	3.8	3.9	4.0
45	3.4	3.2	3.4	3.2
46	4.2	5.2	5.4	5.7
47	5.0	4.8	5.0	5.2
48	4.3	3.9	4.2	4.1
49	3.5	3.4	3.5	3.4
50	3.0	2.9	3.0	2.9
51	4.6	4.3	4.2	4.6
52	3.8	3.6	3.7	3.7
53	2.8	2.8	2.8	2.8
54	2.3	2.2	2.1	1.9
55	1.2	1.3	1.1	0.6
56	5.4	4.9	5.1	5.4
57	4.4	4.2	4.5	4.5
58	3.6	3.4	3.5	3.5
59	2.8	2.8	2.7	2.7
60	2.0	2.1	1.9	1.7
61	4.9	5.0	5.1	5.5
62	4.6	4.2	4.4	4.5
63	3.6	3.5	3.6	3.6
64	3.0	2.8	2.9	2.7
65	2.3	2.4	2.2	2.2
66	4.7	4.4	4.7	4.7
67	3.7	3.5	3.7	3.6
68	2.8	2.8	2.8	3.9
69	2.1	2.2	2.0	3.2
70	1.6	1.7	1.6	5.8
71	5.8	5.2	5.9	5.8
72	4.6	4.2	4.6	4.5
73	3.7	3.4	3.6	3.5
74	3.0	2.9	2.9	2.9
75	2.1	1.9	2.1	1.8
76 (St.)	3.8	3.6	3.7	3.7
77 (St.)	3.2	3.3	3.2	3.2
78 (St.)	1.9	1.8	No Cq	1.3
79 (St.)	1.4	1.6	1.3	1.4
80 (St.)	0.9	1.3	0.8	0.8

1 = 1st run by the same method, 2 = 2nd run by the same method, St. = standard curve samples.

Table S3: qPCR results expressed as log_{10} genome copy numbers

	Method 1 (DTU)	
	Log_{10} genome copies/mL 1	Log_{10} genome copies/mL 2
Sample no.		
1	9.1	9.1
2	8.5	8.5
3	7.1	7.0
4	6.3	6.2
5	5.5	5.5
6	8.9	9.0
7	7.9	7.9
8	7.1	7.0
9	6.3	6.3
10	5.6	5.6
11	8.5	8.3
12	8.3	8.3
13	7.3	7.3
14	6.6	6.5
15	5.9	5.7
16	9.3	9.4
17	8.2	8.2
18	7.1	6.9
19	6.4	6.2
20	5.2	5.5
21	8.0	8.0
22	7.2	7.3
23	6.1	6.1
24	5.5	5.4
25	5.1	4.9
26	7.3	7.3
27	6.5	6.4
28	5.5	5.5
29	4.5	4.4
30	4.0	4.1
31	8.3	8.2
32	7.4	7.4
33	6.3	6.2
34	5.4	5.5
35	4.6	4.6
36	8.6	8.5
37	7.3	7.4
38	6.6	6.4

39	5.6	5.6
40	4.9	4.8
41	8.7	8.8
42	7.8	7.5
43	6.7	6.7
44	5.8	5.6
45	4.7	4.8
46	7.8	7.5
47	6.8	7.0
48	5.9	5.9
49	5.1	4.9
50	4.6	4.5
51	6.7	6.5
52	5.5	5.4
53	4.5	4.5
54	3.7	3.5
55	2.6	2.3
56	7.3	7.2
57	6.4	6.3
58	5.5	5.3
59	4.3	4.4
60	3.4	3.5
61	7.2	7.2
62	6.4	6.4
63	5.3	5.3
64	4.4	4.6
65	3.9	3.9
66	6.4	6.3
67	5.4	5.3
68	4.4	4.4
69	3.4	3.7
70	2.7	2.7
71	7.6	7.3
72	6.4	6.2
73	5.3	5.3
74	4.4	4.4
75	3.4	3.1
76 (St.)	5.3	5.3
77 (St.)	4.9	4.8
78 (St.)	3.4	3.0
79 (St.)	2.6	2.6
80 (St.)	2.7	2.1

1 = 1st run by the same method, 2 = 2nd run by the same method, St. = standard curve samples.

Table S4: Virus infectivity assay results in different cell types

	**PBMCs** (FLI) log_{10} HAD_{50}/mL	**PAMs** (WBVR) log_{10} HAD_{50}/mL	**PAMs** (DTU) log_{10} $TCID_{50}$/mL	**WSL** (FLI) log_{10} $TCID_{50}$/mL	**MARC** (WBVR) log_{10} $TCID_{50}$/mL	**MARC** (DTU) log_{10} $TCID_{50}$/mL	**MARC** (DTU) log_{10} $TCID_{50}$/mL
Sample no.							
1	7.5	8.1	8.6	5.8	5.6	5.7	5.3
2	7.0	7.2	7.2	5.0	5.9	4.6	4.4
3	5.3	5.6	5.3	4.5	3.4	3.7	3.9
4	5.0	5.4	4.8	2.8	2.6	2.9	2.9
5	4.0	3.9	3.7	Negative	1.6	1.8	2.5
6	7.5	8.4	8.6	Negative	6.2	6.4	6.2
7	7.5	7.4	6.7	5.5	4.2	5.1	5.8
8	5.5	5.6	5.0	5.8	3.4	4.3	3.6
9	4.5	4.4	5.1	2.8	3.9	2.3	3.6
10	4.5	4.2	3.7	1.8	2.4	2.3	2.3
11	7.5	8.4	8.1	4.5	5.4	6.0	6.0
12	6.5	7.4	6.7	5.0	4.2	5.1	5.1
13	5.0	5.6	5.8	3.8	3.4	3.9	3.7
14	5.3	5.2	5.5	3.3	2.6	3.2	3.6
15	3.5	4.2	4.4	Negative	1.6	2.9	2.3
16	7.3	7.9	8.6	5.5	4.9	5.7	5.8
17	5.8	6.9	6.5	7.5	3.9	4.4	5.0
18	4.5	5.4	6.0	3.8	2.6	3.2	3.7
19	3.5	4.4	5.1	3.0	1.4	2.5	2.7
20	2.8	3.4	3.7	2.5	0.9	2.3	2.3
21	5.3	6.4	6.2	6.0	4.2	5.0	5.1
22	4.3	6.4	5.3	4.0	3.4	3.7	3.7
23	2.8	3.9	4.3	3.0	1.2	2.3	2.1
24	3.3	2.9	3.7	3.5	0.9	1.6	1.8
25	Negative	2.9	3.0	Negative	Negative	1.6	2.0
26	4.5	6.2	5.8	4.8	3.9	3.9	4.3
27	3.8	5.4	4.6	3.5	1.9	2.5	2.9
28	3.3	3.4	3.6	Negative	0.9	1.6	2.1
29	Negative	2.6	1.0	2.3	0.9	Negative	Negative
30	Negative	0.9	Negative	Negative	Negative	Negative	Negative
31	6.8	6.9	6.5	5.8	5.4	5.8	5.1
32	4.0	6.4	5.3	Negative	2.9	3.7	4.1
33	4.5	4.6	3.7	3.0	0.9	2.3	2.3
34	2.5	2.2	2.1	2.8	0.9	2.3	1.8
35	Negative	0.9	1.3	Negative	Negative	1.8	2.1
36	7.3	7.4	7.1	5.3	5.7	5.8	6.0
37	5.5	6.2	5.7	4.5	2.9	3.9	3.9
38	4.5	4.4	5.0	Negative	1.9	3.7	3.2

39	1.8	3.4	3.6	Negative	1.4	2.3	2.1
40	2.0	1.2	Negative	1.8	Negative	1.0	1.0
41	7.5	7.6	7.4	6.3	5.7	5.7	6.4
42	6.3	6.2	6.4	6.0	3.2	4.3	4.8
43	4.3	4.6	5.0	4.0	1.2	2.5	3.0
44	2.8	1.9	2.7	2.5	Negative	Negative	1.0
45	Negative	1.2	1.3	6.0	Negative	Negative	1.0
46	5.8	6.4	6.4	4.8	4.2	5.3	5.1
47	4.5	5.4	5.1	5.3	2.6	3.7	3.7
48	3.3	3.6	3.9	4.3	1.4	2.1	2.5
49	2.0	2.4	2.3	2.0	0.9	2.1	1.8
50	1.8	1.2	Negative	3.0	0.9	Negative	1.6
51	4.3	5.2	5.1	3.5	2.2	3.2	3.2
52	2.8	4.2	2.5	3.8	0.9	2.1	2.0
53	Negative	1.9	1.0	Negative	Negative	Negative	Negative
54	Negative	Negative	Negative	Negative	Negative	Negative	Negative
55	Negative	1.2	Negative	Negative	Negative	Negative	Negative
56	5.0	6.2	5.7	3.8	3.9	3.4	3.7
57	4.0	4.4	3.4	3.3	2.2	1.8	2.3
58	3.5	2.6	2.3	Negative	Negative	1.8	1.0
59	Negative	1.3	Negative	2.5	Negative	1.6	1.6
60	Negative	Negative	Negative	Negative	Negative	1.0	Negative
61	5.5	5.9	6.4	6.3	4.4	4.1	4.4
62	4.3	4.7	3.7	3.8	1.6	3.0	2.9
63	2.8	2.4	2.9	Negative	Negative	1.6	1.8
64	Negative	Negative	3.6	Negative	Negative	1.8	2.7
65	Negative	0.9	Negative	Negative	Negative	Negative	Negative
66	4.8	5.2	5.8	3.5	2.2	3.0	3.4
67	3.5	3.9	3.9	2.8	0.9	3.0	2.3
68	Negative	3.5	2.5	Negative	0.9	1.0	1.3
69	Negative	1.4	Negative	Negative	1.2	Negative	1.8
70	Negative	1.4	Negative	Negative	Negative	Negative	1.0
71	5.0	5.9	6.5	6.0	4.2	4.3	4.4
72	4.0	4.6	5.1	2.0	1.4	3.0	2.9
73	2.8	3.2	3.7	2.5	0.9	2.1	2.0
74	2.8	Negative	1.0	3.0	Negative	Negative	1.6
75	Negative	Negative	Negative	Negative	0.9	Negative	Negative
76 (St.)	3.0	3.4	3.2	3.8	1.4	3.0	2.5
77 (St.)	Negative	1.6	1.6	Negative	Negative	Negative	Negative
78 (St.)	Negative	Negative	Negative	Negative	Negative	Negative	Negative
79 (St.)	Negative	Negative	Negative	Negative	Negative	Negative	Negative
80 (St.)	Negative	Negative	1.0	Negative	Negative	Negative	Negative

PAMs = pulmonary alveolar macrophages, PBMCs = peripheral blood mononuclear cells, WSL = wild boar lung cells, MARC = MARC145 cells, St. = standard curve samples.

Figure S1: Standard curves applied to determine $TCID_{50}$ equivalents (in Excel)

	Virus dilution	Virus titre log_{10} $TCID_{50}$/mL	Method 4 (Cq 1) Cq-value	Method 4 (Cq 2) Cq-value	Method 5 (Cq 1) Cq-value	Method 5 (Cq 2) Cq-value
	1/40	3.7	24.0	25.2	24.0	25.0
	1/160	3.2	26.1	26.5	26.0	27.1
	1/2560	2.0	31.5	33.9		
	1/10240	1.4	33.6	34.9	33.1	33.6
	1/40960	0.8	35.4		35.0	36.0
Slope:			-0.249	-0.210	-0.262	-0.268
Intercept:			9.718	8.890	10.005	10.404
$R^{2:}$			0.994	0.969	0.999	0.999

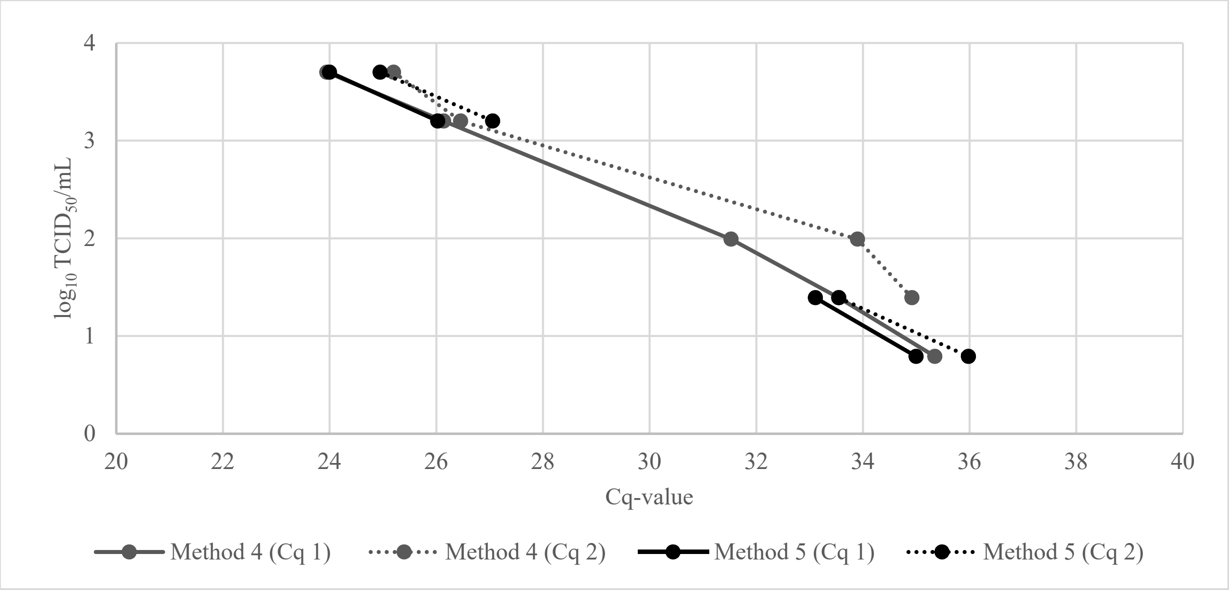

Figure S2: Standard curve applied to determine absolute copy numbers (in MxPro)

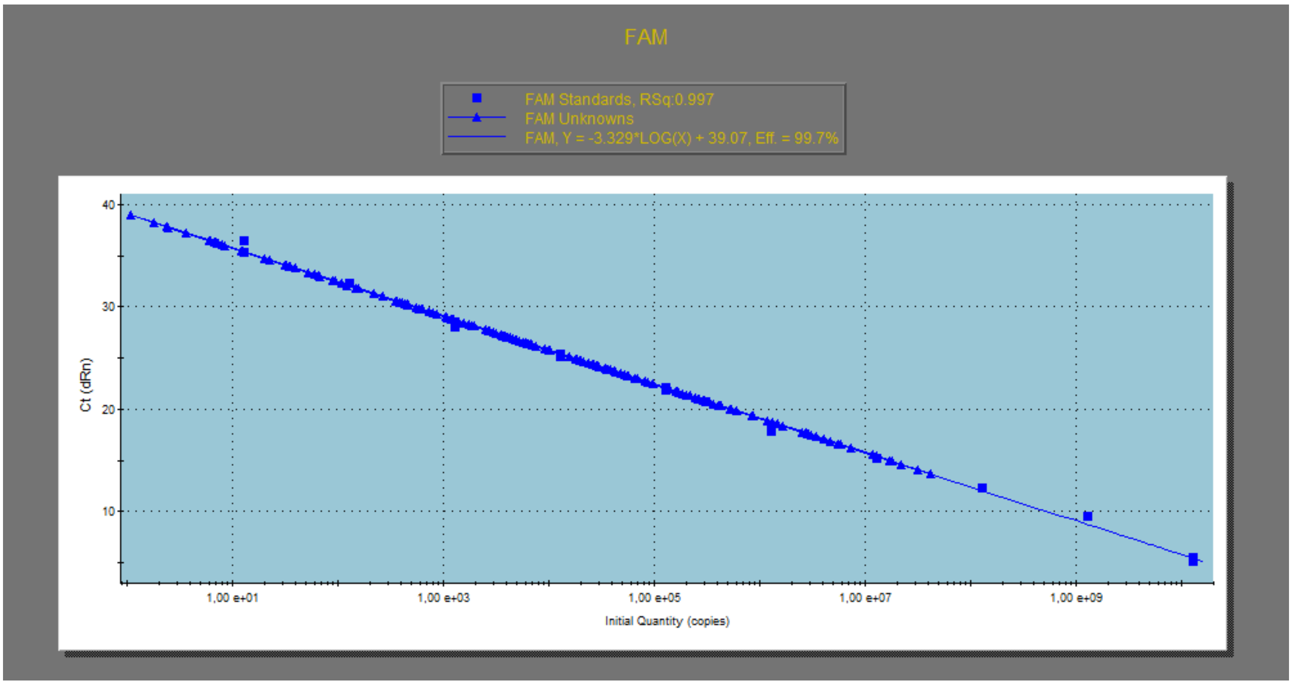

Supplement: Supplementary file 1 [file pathogens-11-00325-s001.zip › pathogens-1601006-supplementary.pdf]
